# Genetic parameters for tick counts across months for different tick species and anatomical locations in South African Nguni cattle

**DOI:** 10.1007/s11250-017-1336-2

**Published:** 2017-07-08

**Authors:** N.O Mapholi, A. Maiwashe, O. Matika, V. Riggio, C. Banga, M.D. MacNeil, V. Muchenje, K. Nephawe, K. Dzama

**Affiliations:** 1 Department of Life and Consumer Sciences, University of South Africa, Private Bag X6, Florida, 1710 South Africa; 2 Agricultural Research Council, Private Bag X2, Irene, 0062 South Africa; 3 Department of Animal Sciences, University of the Free State, PO Box 339, Bloemfontein, 9300 South Africa; 4 The Roslin Institute and R(D)SVS, University of Edinburgh, Edinburgh, UK; 5 Delta G, 145 Ice Cave Road, Miles City, MT 59301 USA; 6 University of Fort Hare, Private Bag X 1314, Alice, South Africa; 7 Department of Animal Sciences, Faculty of Science, Tshwane University of Technology (TUT), P/Bag X680, Pretoria, 0001 South Africa; 8 Department of Animal Science, University of Stellenbosch, P Bag X1, Matieland, 7602 South Africa

**Keywords:** Tick resistance, Tick count, Heritability, Correlation

## Abstract

The objective of the study was to characterise genetic parameters across months for different tick species and anatomical locations in South African Nguni cattle. Tick counts were conducted monthly, over a 2-year period, on 586 Nguni cattle under natural infestation, from four herds located in different provinces of South Africa. The counts were recorded for six species of ticks (*Amblyomma hebraeum, Rhipicephalus evertsi evertsi, Rhipicephalus decoleratus* and *microplus (Boofilids), Rhipicephalus appendiculatus, Rhipicephalus simus and Hyalomma marginatum*) attached on eight anatomical locations on the animals and were summed by species and anatomical location. Heritability estimates, phenotypic and genetic correlations were estimated on a monthly basis using mixed linear models, fitting univariate and bivariate sire models. Fixed effects considered were location, sex, year and age as a covariate. Tick counts were higher in the hot months, and *A. hebraeum* was the most dominant tick species. Heritability estimates for tick count varied by month and trait and ranged from 0 to 0.89. Genetic correlations were mostly positive, and low to high, with some negative correlations with high standard error. Phenotypic correlations were low to moderate. In general, high genetic correlations were observed between whole body count and the anatomical location counts, suggesting that it may not be necessary to conduct whole body counts. Counts from the belly and perineum appeared to be the most suitable surrogate traits for whole body count. These findings provide useful information for developing strategies for the practical implementation of genetic selection, as a supplement to the traditional tick control measures.

**Electronic supplementary material:**

The online version of this article (doi:10.1007/s11250-017-1336-2) contains supplementary material, which is available to authorized users.

## Introduction

Economic losses in livestock production due to ticks and tick-borne diseases have long been a major concern to livestock producers in tropical and sub-tropical regions including South Africa (Seifert 1984; Passafaro et al. 2015; Mota et al. 2016). The most economically important tick genera affecting livestock production in Southern Africa are *Rhipicephalus* (includes the genus formerly known as *Boophilus*), *Amblyomma* and *Hyalomma* (Marufu et al. 2011; Nyangiwe et al. 2013; Mapholi et al. 2013, 2016). These tick genera have an impact on animal productivity directly through heavy infestation and indirectly through transmission of tick-borne diseases (Dold and Cocks 2001; Ghosh et al. 2006). A large portion of the cost associated with ticks is from the implementation of control measures, mainly chemical acaricides, to reduce tick loads (de Castro 1997; Porto-Neto et al. 2011). Increasing prices of acaricides and resistance of ticks to such chemicals are increasing problems and real economic burdens to the livestock producers (Mukhebi et al. 1992; Rajput et al. 2006; Kabi et al. 2014; Muyobela et al. 2015). Hence, there is a pressing need for alternative ways to reduce tick infestations in livestock. One possibility is the identification and use of cattle that are naturally resistant to ticks (Hayward 1981).

Host resistance refers to an animal’s ability to prevent maturation of large numbers of ticks and having immunity to tick-borne diseases (Roberts 1968). Such resistance may be measured by counting or scoring the number of ticks on the animal following either artificial or natural infestation (Porto-Neto et al. 2011). Use of artificial infestation with known numbers of tick larvae, followed by the counting of engorging adult females, has been suggested as the most appropriate method to measure tick resistance (Regitano et al. 2006). Host resistance to ticks is under genetic control (Hewetson 1972) and genetic variation in tick resistance varies within and among breeds (Seifert 1971; Utech et al. 1978; Utech and Wharton 1982; Prayaga et al. 2009; Mapholi et al. 2013). Zebu cattle (*Bos indicus*) in Brazil and Australia has greater tick resistance when compared to European cattle (*Bos taurus*) (Utech and Wharton 1982; Madalena et al. 1990; Frisch and O’Neill 1998; Mwangi et al. 1998; Wambura et al. 1998; da Silva et al. 2007). Indigenous breeds in South Africa have also been reported to be more resistant to ticks than European cattle (Spickett et al. 1989; Scholtz et al. 1991; Latif 2006). Muchenje et al. (2008) also reported that Nguni cattle were less susceptible to ticks when compared to Bonsmara (composite breed from *Bos taurus* × *Bos indicus*). Resistance of cattle to ticks is heritable and responsive to selection (Burrow 2001; Turner et al. 2010). Heritability estimates for resistance to ticks range from 0.05 to 0.42 (Wharton et al. 1970; Burrow 2001; Prayaga and Henshall 2005; Peixoto et al. 2008; Prayaga et al. 2009; Budeli et al. 2009; Porto-Neto et al. 2014; Ayres et al. 2013). Reliable estimates of genetic parameters are a prerequisite for using selection to genetically improve any trait. Thus, the main objective of the current study was to characterise genetic parameters for tick counts across months for different tick species and anatomical locations in South African Nguni cattle.

## Materials and methods

### Experimental cattle

Tick count data were collected from 586 randomly selected Nguni cattle (61 males and 525 females) over a 2-year period from four different herds in different agro-climatic zones (locations): Agricultural Research Council (ARC) Loskop Research Farm located in the Limpopo Province of South Africa (*n* = 124), ARC-Roodeplaat Research Farm located in Gauteng Province (*n* = 143), Mukhuthali Nguni Community Farm located in the Kwa-Zulu Natal Province (*n* = 224) and the University of Fort Hare Farm in Alice located in the Eastern Cape Province (*n* = 95). Ages of the cattle and their physiological status varied in each location, with age ranging from 2.5 to 17 years. Limited pedigree information was available, with 806 animals over three generations. Animals were exposed to natural tick infestation at all four farms. Counts and identification of tick species were conducted every month from May 2012 to April 2014. All animals were spray dipped with a flumethrin pour-on formulation “Drastic Deadline®” immediately after the tick count data collection each month.

### Tick count data collection

Tick counts in all the four herds were recorded on a monthly basis by the same group of trained technicians throughout the experiment. Two people conducted counts on an animal at a time, with each technician counting and identifying tick species on half of the body. Adult ticks were counted on eight anatomical locations (head, excluding within the ears; within the ears, neck and gullet, back, legs and belly, including the udder or testicles; perineum and tail, including underneath the tail) and recorded by species (*Amblyomma hebraeum, Rhipicephalus evertsi evertsi, Rhipicephalus decoleratus* and *microplus* (*Boofilids*) spp., *Rhipicephalus appendiculatus, Rhipicephalus simus and Hyalomma marginatum*). Total counts by species and anatomical location were also considered, resulting in 63 measured phenotypes or traits. See the detailed description of the 63 traits (Mapholi et al. 2016). After inspecting the raw data, nine of these traits (Table 1) were selected for further analysis, based on the availability of non-zero counts.

**Table 1:** Abbreviations and full identification for each of the analysed tick count traits

Trait	Trait full name
Anatomical location of the animal	
Whole body	Whole body tick count
Belly	Total tick count on the belly
Perineum	Total tick count on the perineum
Tail	Total tick count on the tail
Tick species per anatomical location of the animal	
*A. hebraeum* on perineum	Total count of *Amblyomma hebraeum* ticks on the perineum
Boofilids on perineum	Total count of Boofilids ticks on the perineum
Tick species	
*A. hebraeum*	Total count of *Amblyomma hebraeum* tick count on the whole body
*R. evertsi evertsi*	Total count of *Rhipicephalus evertsi evertsi* tick count on the whole body
Boofilids	Total count of Boofilds tick count on the whole body

### Statistical analyses

Since tick counts were skewed, data were log-transformed so that the distribution could approximate normality (see Supplementary Fig. 1 for an example). All subsequent analyses were then carried out on the transformed phenotypes. Preliminary analyses were conducted using the PROC GLM procedure (SAS 2010) to determine environmental factors influencing tick count by fitting the following fixed effects model:

1 $$ {Y}_{\mathrm{ijkn}}=\mu +{L}_i+{R}_j+{S}_k+\mathrm{bA}+{e}_{\mathrm{ijkn}} $$

where *Y*_ijkln_ is the monthly log transformed tick count, *μ* is the overall mean, *L*_*i*_ is the effect of the *i*th location (farm) (*i* = 1, 2, 3, 4), *R*_*j*_ is the effect of the *j*th year (*j* = 1, 2), *S*_*k*_ is the effect of the *k*th sex (*k* = 1, 2), *b* is the regression coefficient of age of the animal on tick count, A is the age of the animal and *e*_ijkln_ is the random residual error.

### Genetic parameter estimates

Variance components and heritabilities for log-transformed tick counts were estimated by univariate analysis fitting a sire model in ASREML software (Gilmour et al. 2002). The sire model was preferred due to the fact that the pedigree data was incomplete but with enough sires. The following model was used:

2 $$ y=\mathrm{Xb}+\mathrm{Zs}+\mathrm{Wpe}+\mathrm{e} $$

where *y* is a vector of observations (monthly log-transformed tick count), *b*, *s*, *pe* and *e* are the vectors of fixed effects (according to Eq. 1), random additive sire genetic effects, permanent environmental effects due to the animals and residuals, and *X*, *Z* and *W* are incidence matrices relating the fixed and random effects respectively to *y*. Random effects were assumed to be normally distributed with sire ∼*N* (0, *Aσ* ^2^_*s*_), permanent environment ∼*N* (0, *Iσ* ^2^_pe_), residual ∼*N* (0, *Iσ* ^2^_*e*_), where *A* is a numerator relationship matrix and *I* is an identity matrix of order equal to the number of animals and records, *σ* ^2^_s_, *σ* ^2^_pe_ and *σ* ^2^_e_ are the sire, permanent environmental and residual variances, respectively. The relationship matrix was constructed using pedigree. The narrow-sense heritability (*h* ^2^) was calculated as follows: sire model *h* ^2^ = 4*σ* ^2^_s_/(*σ* ^2^_s_ *+ σ* ^2^_pe_ + *σ* ^2^_e_). A series of bivariate analyses were used, fitting the same model as above, to estimate genetic and phenotypic correlations among the traits of interest, considering mostly the traits with significant heritability estimates from the univariate analysis.

## Results

**Fig. 1:**
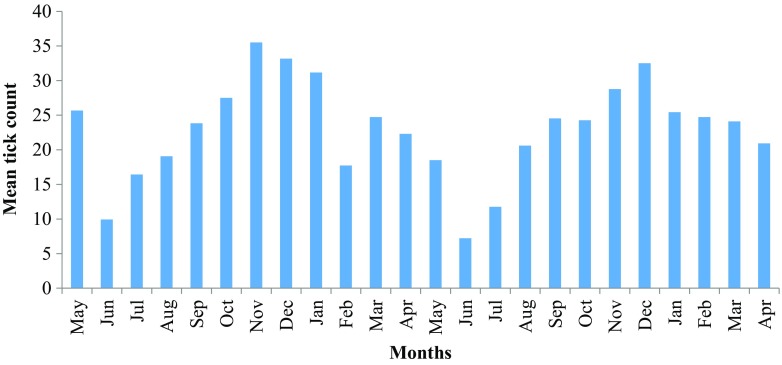
Distribution of whole body tick count in Nguni cattle over 2 years

Tick counts were higher in the hot months and *A. hebraeum* was the most dominant tick species. The mean monthly whole body tick counts were lowest (8.4 ± 6.1) in June (Winter) and highest (32.1 ± 23.4) in November (Summer); similar trends were observed for the other traits analysed (Table 2), except for Boofilids (total tick count and on the perineum) which had highest mean values in March. This same trend was also observed in the plot for mean tick counts in the full dataset (Fig. 1).

**Table 2:** Mean and standard deviations for monthly tick count in Nguni cattle

Month/trait	February	March	May	June	July	August	September	November
Number of records	1102	1102	946	1008	1008	1008	1102	1102
Anatomical location of the animal								
Whole body	21.3 ± 15.9	24.4 ± 12.0	21.4 ± 12.7	8.4 ± 6.1	13.8 ± 9.6	19.9 ± 11.2	24.2 ± 12.5	32.1 ± 23.4
Belly	4.2 ± 5.4	4.9 ± 4.8	4.7 ± 4.3	2.3 ± 2.7	4.4 ± 5.3	4.5 ± 4.6	4.8 ± 4.0	7.5 ± 7.4
Perineum	4.8 ± 5.6	6.5 ± 5.9	4.0 ± 4.1	1.0 ± 2.0	2.3 ± 2.8	4.0 ± 3.6	5.7 ± 4.9	7.5 ± 7.4
Tail	8.1 ± 6.6	9.3 ± 6.1	6.2 ± 4.4	2.4 ± 3.0	3.5 ± 4.1	6.8 ± 5.5	10.2 ± 6.5	9.8 ± 8.0
Tick species per anatomical location of the animal								
*Amblyomma hebraeum* on perineum	2.3 ± 2.9	2.9 ± 3.4	2.6 ± 3.0	0.5 ± 1.4	1.3 ± 2.3	2.5 ± 2.8	4.0 ± 4.2	4.0 ± 4.3
Boofilids on perineum	1.2 ± 3.1	2.3 ± 4.3	0.6 ± 2.5	0.1 ± 0.6	0.2 ± 0.9	0.3 ± 1.2	0.4 ± 1.6	1.5 ± 5.2
Tick species								
*Amblyomma hebraeum*	8.1 ± 6.8	8.9 ± 6.3	9.1 ± 6.4	4.2 ± 4.3	6.7 ± 6.9	10.2 ± 8.6	11.5 ± 8.6	12.3 ± 9.5
*Rhipicephalus evertsi evertsi*	4.6 ± 1.9	5.6 ± 4.7	3.6 ± 3.7	1.2 ± 2.3	2.7 ± 4.0	4.1 ± 4.4	6.3 ± 5.0	6.2 ± 5.1
Boofilids	4.9 ± 9.5	5.6 ± 7.5	3.5 ± 7.9	1.1 ± 2.0	1.9 ± 2.7	2.2 ± 2.8	2.3 ± 3.3	5.4 ± 13.2

In most of the traits analysed, all fixed effects with the exception of sex were significant (*p* < 0.05). Age fitted as covariate was also significant in most traits.

### Heritability estimates

Monthly heritability estimates for tick counts on different body locations (i.e. whole body, perineum, belly and tail) were low to moderate, ranging from 0 to 0.58 (Table 3). For whole body tick count, the estimates were significant in March and May, whereas on the belly, they were more spread across months, between March and August. Significant estimates for the perineum tick count were observed in June and July, whereas on the tail, the only significant estimate was in July. The proportion of variation explained by the permanent environment due to the animal (pe) was zero to low across all traits (Table 3).

**Table 3:** Heritability estimates for tick count by anatomical location in Nguni cattle

	Whole body		Belly		Perineum		Tail	
	*h* ^2^ ± se	pe ± se	*h* ^2^ ± se	pe ± se	*h* ^2^ ± se	pe ± se	*h* ^2^ ± se	pe ± se
February	0.00	0.00	0.11 ± 0.11	0.00	0.04 ± 0.09	0.00	0.00	0.00
March	0.40 ± 0.16	0.08 ± 0.04	0.40 ± 0.16	0.06 ± 0.04	0.06 ± 0.10	0.00	0.21 ± 0.12	0.00
May	0.57 ± 0.22	0.13 ± 0.04	0.32 ± 0.16	0.00	0.16 ± 0.12	0.00	0.25 ± 0.17	0.04 ± 0.05
June	0.00	0.18 ± 0.05	0.14 ± 0.13	0.00	0.58 ± 0.22	0.00	0.00	0.09 ± 0.05
July	0.01 ± 0.11	0.13 ± 0.05	0.33 ± 0.17	0.00	0.38 ± 0.19	0.02 ± 0.04	0.36 ± 0.18	0.00
August	0.15 ± 0.14	0.00	0.42 ± 0.20	0.00	0.22 ± 0.15	0.00	0.22 ± 0.16	0.00
September	0.00	0.08 ± 0.04	0.00	0.01 ± 0.04	0.00	0.00	0.12 ± 0.12	0.06 ± 0.04
November	0.00	0.00	0.08 ± 0.11	0.02 ± 0.04	0.33 ± 0.17	0.05 ± 0.04	0.00	0.00

*h* ^*2*^ heritability estimate, *se* standard error, *pe* proportion of phenotypic variance due to the permanent environment

When considering the heritability estimates for tick count for different tick species, only one estimate was significant for *A. hebraeum* (May) and one for *R. evertsi evertsi* (September), with no effects of the permanent environment due to the animal (Table 4). However, in the heritability estimates for tick species on the perineum, low to high estimates (from 0 to 0.89) were observed, with the highest estimate being for total *Boofilids* ticks found on the perineum in February (Table 5). Heritability estimates for months (i.e. January, April, October, and December) where all estimates were close zero or not significant were not included in the Tables.

**Table 4:** Heritability estimates for tick count by tick species in Nguni cattle

	*Amblyomma hebraeum*		*Rhipicephalus evertsi evertsi*		Boofilids	
	*h* ^2^ ± se	pe ± se	*h* ^2^ ± se	pe ± se	*h* ^2^ ± se	pe ± se
February	0.00	0.00	0.00	0.01 ± 0.04	0.23 ± 0.14	0.00
March	0.02 ± 0.09	0.03 ± 0.04	0.11 ± 0.10	0.06 ± 0.04	0.00	0.00
May	0.55 ± 0.22	0.06 ± 0.05	0.15 ± 0.13	0.00	0.13 ± 0.11	0.00
June	0.15 ± 0.14	0.01 ± 0.05	0.00	0.00	0.10 ± 0.12	0.00
July	0.02 ± 0.10	0.02 ± 0.05	0.19 ± 0.15	0.00	0.00	0.00
August	0.28 ± 0.16	0.00	0.00	0.00	0.16 ± 0.13	0.00
September	0.09 ± 0.11	0.02 ± 0.04	0.38 ± 0.17	0.01 ± 0.04	0.02 ± 0.07	0.00
November	0.03 ± 0.09	0.00	0.02 ± 0.04	0.02 ± 0.08	0.22 ± 0.13	0.00

*h* ^*2*^ heritability estimate, *se* standard error, *pe* proportion of phenotypic variance due to the permanent environment

**Table 5:** Heritability estimates for tick count by tick species on the perineum body location in Nguni cattle

	*Amblyomma hebraeum*		*Boofilids*	
	*h* ^2^ ± se	pe ± se	*h* ^2^ ± se	pe ± se
February	0.06 ± 0.09	0.00	0.89 ± 0.23	0.04 ± 0.04
March	0.02 ± 0.08	0.00	0.00	0.00
May	0.05 ± 0.09	0.00	0.32 ± 0.15	0.00
June	0.53 ± 0.22	0.00	0.00	0.00
July	0.00	0.08 ± 0.12	0.75 ± 0.24	0.00
August	0.20 ± 0.14	0.04 ± 0.04	0.61 ± 0.23	0.00
September	0.00	0.00	0.32 ± 0.15	0.00
November	0.13 ± 0.12	0.00	0.23 ± 0.15	0.12 ± 0.04

*h* ^*2*^ heritability estimate, *se* standard error, *pe* proportion of phenotypic variance due to the permanent environment

**Fig. 2:**
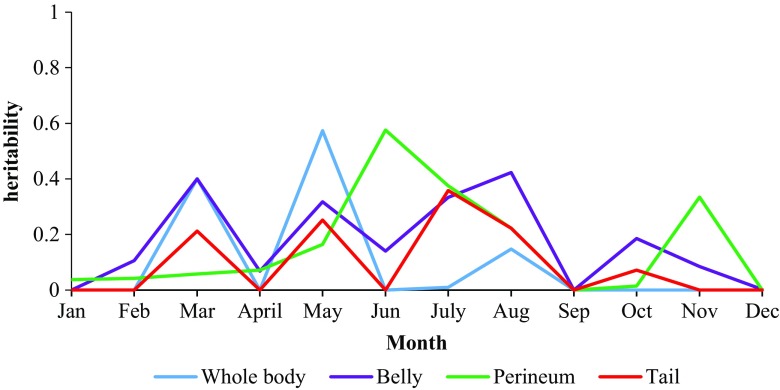
Monthly heritability trends of tick count for different anatomical locations in Nguni cattle

**Fig. 3:**
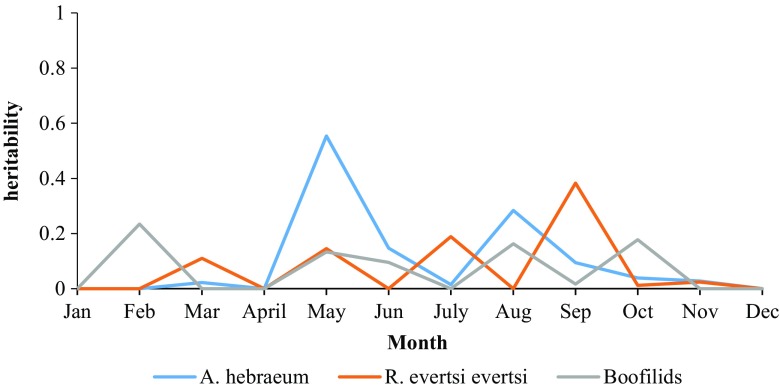
Monthly heritability trends of tick count for different tick species in Nguni cattle

**Fig. 4:**
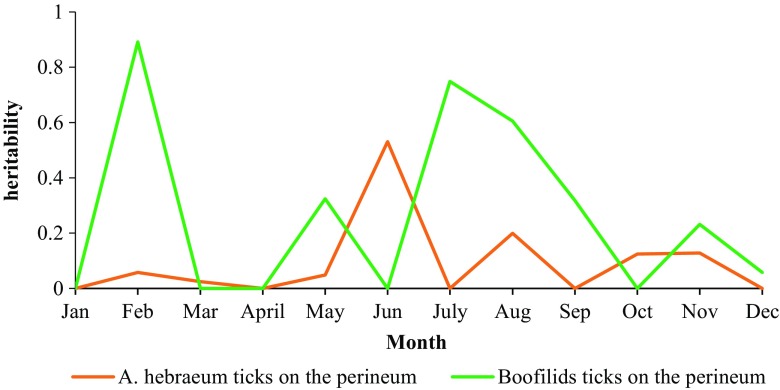
Monthly heritability trends of tick count for different tick species located on the perineum in Nguni cattle

There was no observed trend across monthly heritabilities, as shown in Figs. 2, 3 and 4.

### Genetic and phenotypic correlations

Moderate to high genetic correlations were estimated across the traits analysed. Estimates close to unity were observed between whole body tick count and belly in March and May, between perineum and Boofilids on perineum in July and November, and between total tick count of Boofilids and Boofilids on the perineum in February. Surprisingly, high genetic correlation at unity was also observed between the total tick count on the belly and Boofilids on perineum in May. Moderate to high correlations were also estimated between Boofilids on the perineum and *A. hebraeum* (in May) and *R. evertsi evertsi* (in September) (Table 6). Few negative genetic correlations were observed; however, they were not significant except for that between total tick count on the belly and on the tail in May (−0.39). Phenotypic correlations were mostly positive and low to moderate, ranging from 0.01 to 0.69. However, few negative phenotypic correlations with high standard error were also observed (Table 6).

**Table 6:** Genetic (above) and phenotypic (below) correlations across months and tick count traits in Nguni cattle

	Whole body	Belly	Perineum	Tail	*Amblyomma hebraeum*	*Rhipicephalus evertsi evertsi*	*Boofilids*	*A. hebraeum* on perineum	*Boofilids* on perineum
February									
Boofilids	–	–	–	–	–	–	–	–	1.00 ± 0.05 0.62 ± 0.03
March									
Belly	1.00 ± 0.07 0.48 ± 0.03	–	–	–	–	–	–	–	–
May									
Whole body	–	–	1.00 ± 0.11 0.56 ± 0.03	0.67 ± 0.21 0.57 ± 0.03	0.89 ± 0.08 0.68 ± 0.02	–	–	–	0.82 ± 0.21 0.28 ± 0.04
Belly	0.96 ± 0.09 0.56 ± 0.03	–	–	0.39 ± 0.33 0.08 ± 0.04	0.79 ± 0.14 0.49 ± 0.03	–	–	–	1.00 ± 0.27 0.56 ± 0.03
Tail	–	–	–	–	0.88 ± 0.13 0.55 ± 0.03	–	–	–	−0.03 ± 0.39 −0.13 ± 0.04
*Boofilids* on perineum	–	–	–	–	0.57 ± 0.26 −0.04 ± 0.05	–	–	–	–
June									
Perineum	–	–	–	–	–	–	–	0.85 ± 0.10 0.74 ± 0.02	–
July									
Belly	–	–	–	0.28 ± 0.36 −0.05 ± 0.04	–	–	–	–	−0.25 ± 0.33 −0.09 ± 0.05
Perineum	–	−0.38 ± 0.38 0.20 ± 0.04	–	−0.74 ± 0.37 −0.06 ± 0.04	–	–	–	–	1.00 ± 0.06 0.35 ± 0.04
Tail	–	–	–	–	–	–	–	–	−0.19 ± 0.32 0.13 ± 0.05
August									
Whole body	–	–	0.27 ± 0.50 0.52 ± 0.03	0.61 ± 0.40 0.61 ± 0.02	−0.22 ± 0.52 0.69 ± 0.03	–	–	–	0.46 ± 0.45 0.09 ± 0.04
Belly	0.87 ± 0.19 0.58 ± 0.03	–	−0.15 ± 0.43 0.18 ± 0.04	0.23 ± 0.44 0.10 ± 0.04	0.34 ± 0.32 0.58 ± 0.03	–	–	–	0.12 ± 0.33 0.05 ± 0.05
Perineum	–	–	–	−0.24 ± 0.49 0.29 ± 0.04	0.06 ± 0.44 0.45 ± 0.03	–	–	–	0.54 ± 0.31 0.30 ± 0.04
Tail	–	–	–	–	−0.08 ± 0.45 0.40 ± 0.04	–	–	–	0.07 ± 0.42 0.05 ± 0.04
*Amblyoma hebraeum*	–	–	–	–	–	–	–	–	0.03 ± 0.37 0.01 ± 0.04
September									
Boofilids on perineum	–	–	–	–	–	0.75 ± 0.24 0.12 ± 0.04	–	–	–
November									
Perineum							0.27 ± 0.41 0.22 ± 0.03		1.00 ± 0.15 0.43 ± 0.03
Boofilids	–	–	–	–	–	–	–	–	0.61 ± 0.30 0.60 ± 0.02

Note that (−) means that the correlations of these traits were not calculated

## Discussion

Having monthly data available for tick count collected at different anatomical locations and for different tick species in Nguni cattle over 2 years presented an opportunity to investigate the tick variation across time, and possibly identify optimal sampling time and more suitable traits. This study demonstrated that tick distribution varies across the year and that there is genetic variation across months in tick count, which varies from low to high depending on the trait. The study also identified positive genetic and phenotypic correlations among tick count at different anatomical locations. However, partitioning of data according to tick species did not seem to allow for enough power to estimate heritabilities and genetic correlations. These results should be considered in the context of the limitations and advantages of field studies (Bishop and Woolliams 2010; Bishop et al. 2012). Although the use of unknown and uncontrolled exposure to infections may lead to reduced power in field studies, however, this does not constitute a fatal flaw in demonstrating host genetic differences in resistance (Bishop and Woolliams 2010). Moreover, the natural mixed infections which characterise field studies better reflects the genetic variation of host resistance and yield results that are more relevant to practical genetic improvement programmes.

Tick counts may indicate an animal’s level of infestation. According to Gonzales et al. (1993) and Passafaro et al. (2015), counts from 1 to 5 indicate mild infestation, 5 to 20 moderate infestation, 20 to 50 high infestation and over 50 a very high infestation, with the ideal conditions for tick development being approximately 28 °C for temperature with 80% of humidity (Monteiro et al. 2009).

In this current study, mean tick counts varied from 7 to 35 depending on month. Other studies have reported mean tick count similar to the current study with highest mean values of around 37 for South African Bonsmara (Budeli et al. 2009) and Belmont Red cattle (Corbet et al. 2006). On the other hand, Turner and Short (1972) have observed higher mean tick counts ranging from 20 to 30 for Afrikaner and Brahman cattle, and from 75 to 100 for Shorthorn cattle under natural infestation in Australia, whereas Ayres et al. (2013) have reported lower mean tick counts (11.6) in Nellore and Nellore × Herford crosses under natural infestation. Although indigenous cattle breeds (such as Nguni and Nellore) are reported to be more resistant to tick infestations than the other breeds, these values are not necessarily an indication of genetic resistance of the different breeds, since they can also be influenced by other factors, such as environment, year, management, dipping and type of infestation. Muchenje et al. (2008) observed higher tick infestations on the non-dipped Nguni steers than on the dipped Nguni steers, which implies that dipping play a role in tick control. Proper breed comparisons would entail breeds to be compared under the same environment and conditions.

Another aspect to consider is the minimum time interval between use of acaricides (which can have a different duration of action) and data collection. For example, Passafaro et al. (2015) performed their counts considering a minimum interval of 120 days after the use of any antiparasitic drug. However, in the current study, the duration of action of the acaracides was not taken into account, as dipping was conducted per strategic routine practice in South Africa. This might be due to the fact that in Southern Africa, ticks are vectors to several diseases.

Low to high heritability estimates were observed in this study, depending on month, anatomical location and tick species. However, not all estimates were significant, which can be partly explained by the size of the data. The reason for the variation in heritability estimates is not clear. Previous heritability estimates in literature were low from 0.09 in Brahman cattle (Porto-Neto et al. 2014), 0.13 in composite breeds (Prayaga and Henshall 2005), 0.15 in Brahman cattle (Prayaga et al. 2009), 0.17 in Bonsmara cattle (Budeli et al. 2009), 0.19 in Braford and Hereford cattle (Cardoso et al. 2015) and 0.21 in a Hereford Shorthorn line (Peixoto et al. 2008). However, other authors have reported higher heritability estimates for tick resistance, including 0.37 in *Bos taurus* dairy breeds (Turner et al. 2010), 0.39 in Shorthorn (Wharton and Roulston 1970), 0.41 in a tropical composite breed (Porto-Neto et al. 2014) and 0.42 in a composite breed of tropical beef cattle (Burrow 2001). There could be a number of reasons for the wide variability in heritability estimates. Low heritability estimates obtained from some of these studies might have been due to different sampling methods or low natural tick infestation challenge in the field. Use of a scoring system for infestation rather than tick counts may also affect heritability estimates due to the subjectivity of this method and difficulty in consistent application across studies (Prayaga and Henshall 2005; Prayaga et al. 2009). Environmental factors that affect the intensity of natural infestations, breed of cattle and immune status of the animal should be accounted for when estimating genetic parameters (Porto-Neto et al. 2011). Season also plays an important role in the prevalence of ticks and could, therefore, influence heritability estimates (Wharton et al. 1970). Higher levels of tick infestation, which normally occur in the hot seasons, appear to elicit more genetic variation in tick resistance. For example, Wharton et al. (1970) observed increased heritability estimates for tick burden in summer and low to zero estimates in the winter season. Budeli et al. (2009) also reported moderate heritability estimates when the mean tick count was ≥25 and suggested that tick count data should be collected when the level of tick infestations is high. Other studies in South Africa (Scholtz et al. 1991; Muchenje et al. 2008; Marufu et al. 2011) also reported higher infestations in the hot and dry seasons and recommended that genetic parameters for tick resistance should be estimated during this time of the year. However, in our study, there is no discernible pattern across the months. May and August, which are relatively cool months, had high heritability estimates, while the hottest month (November) had in general very low estimates. Ayres et al. (2013) also observed higher heritability in Winter. However, there is no obvious explanation for this trend.

Some researchers have emphasised that tick count data should be collected when animals have had sufficient exposure to ticks (higher tick infestation), in order to observe the resistance or susceptibility of the animal (Hewetson 1968; Henshall 2004; Latif 2006). It has been noted that a lack of exposure simply means that individuals do not have the opportunity to express their genotype for resistance, with potentially susceptible individuals being misclassified (Bishop and Woolliams 2010). It therefore appears compelling to strategically collect tick count data for genetic evaluation in the season when ticks are more active. Besides capitalising on the relatively high genetic variation in tick resistance realised during that time of the year, it may also minimise the costs of data collection.

The current study observed high genetic correlations between whole body count and the anatomical location counts, which suggest that the use of other anatomical locations such as belly and perineum as proxies for the whole body count is feasible. This might be due to the fact that both these body locations have softer skin with short hair. However, since the perineum is more accessible than the belly, this could be more convenient for tick counting. High genetic correlations were observed between Boofilids total count and Boofilids on the perineum, indicating that the latter can be a good approximation for Boofilids total count.

## Conclusion

In the current study, genetic parameters were characterised for tick counts across months for different tick species and anatomical locations in South African Nguni cattle. Results show sufficient genetic variation to warrant improvement in tick resistance through selection, thereby complementing other tick control methods. Such genetic variation appears to be expressed more during some months of the year than in others. Results therefore suggest that collection of tick count data for genetic selection should be carried out during those months eliciting the highest genetic variation. Tick counts from either the perineum or belly may be used as reliable indicators of whole body count. However, further studies verifying these results are required, before any recommendations are adopted at national scale.

## Electronic supplementary material

Supplementary Figure 1 (DOCX 343 kb)
