# Supplementary material for: Genetic parameters for tick counts across months for different tick species and anatomical locations in South African Nguni cattle
Source: Trop Anim Health Prod. 2017 Jul 8;49(6):1201–10. doi: 10.1007/s11250-017-1336-2 (PMC5519660; doi:10.1007/s11250-017-1336-2)
Supplement: Supplementary file 1 — (DOCX 343 kb) [file 11250_2017_1336_MOESM1_ESM.docx]

**Supplementary Material**


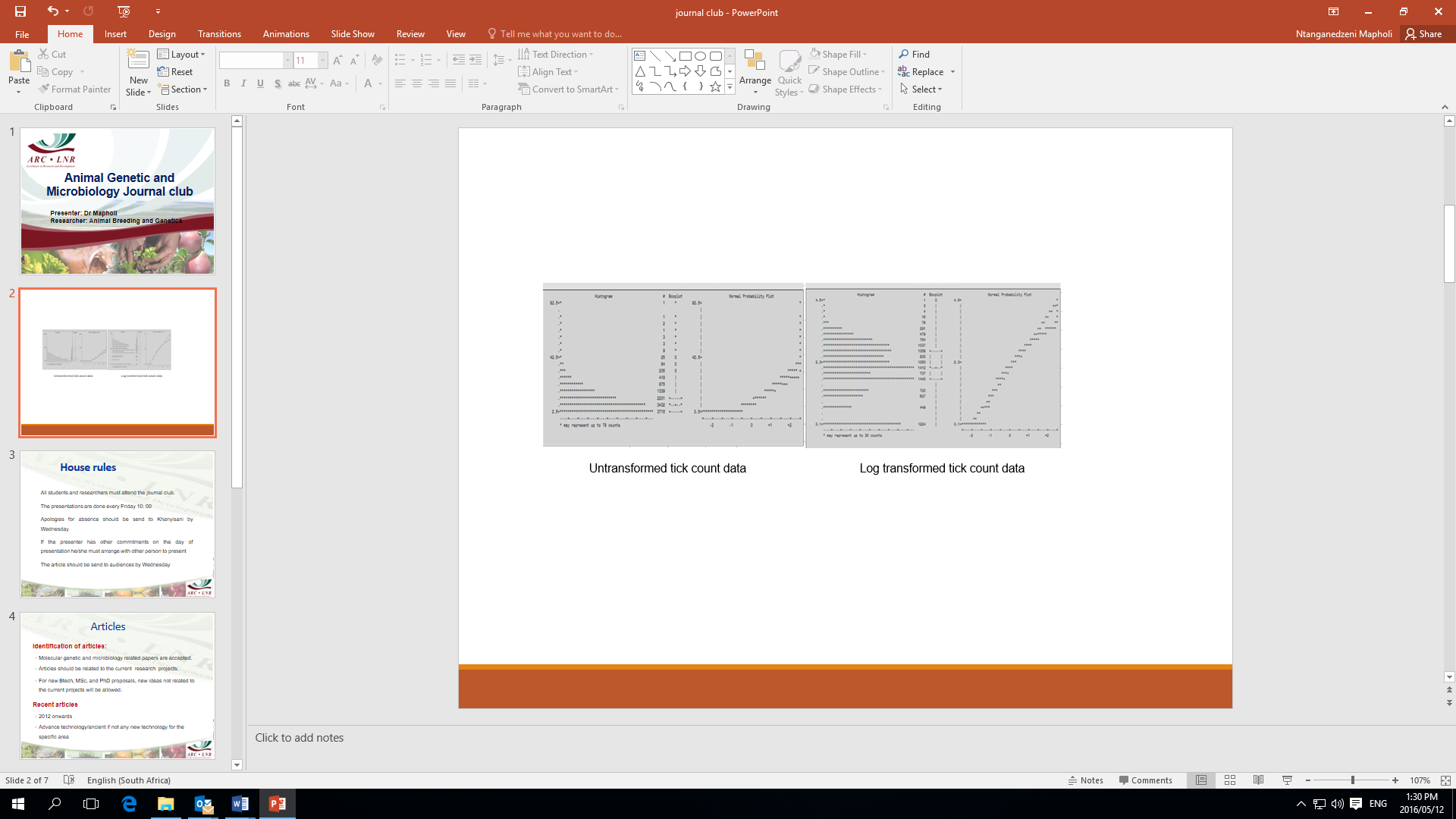


**Supplementary Figure 1:** Normal plots of untransformed and transformed tick count data
